# Cyclophosphamide, Bortezomib, and Dexamethasone and Severe Systolic Heart Failure: A Case Report

**DOI:** 10.7759/cureus.56966

**Published:** 2024-03-26

**Authors:** Sagar Pandey, Ernestine Faye S Tan, Amulya Bellamkonda, Binit Aryal, Madhumati Kalavar

**Affiliations:** 1 Internal Medicine, One Brooklyn Health/Interfaith Medical Center, Brooklyn, USA; 2 Hematology and Oncology, One Brooklyn Health/Interfaith Medical Center, Brooklyn, USA

**Keywords:** chemotherapy-associated cardiotoxicity, dexamethasone, cyclophosphamide, bortezomib, heart failure, multiple myeloma

## Abstract

Multiple myeloma (MM) is a neoplastic proliferation of plasma cells in bone marrow. Pharmacotherapy for the management of patients with MM includes drug classes like proteasome inhibitors, monoclonal antibodies, immunomodulators, alkylating agents, steroids, etc. We present a case of new-onset heart failure with reduced ejection fraction (HFrEF) in a patient with previously normal ejection fraction after treatment with a cyclophosphamide, bortezomib, and dexamethasone (CyBorD) chemotherapeutic regimen. An echocardiogram done after the completion of nine cycles of chemotherapy in a period of about 4.5 months showed severely decreased left ventricular systolic function with an ejection fraction of only 15-20% and grade I diastolic dysfunction. Cardiac catheterization showed no angiographic evidence of vessel occlusion or epicardial disease. HFrEF was managed with the initiation of guideline-directed medical therapy with cardiology clinic follow-up, and the patient was discharged with a plan to start a lenalidomide-based chemotherapeutic regimen with oncology clinic follow-up. It is, therefore, imperative to perform a thorough cardiovascular assessment before initiation of chemotherapy, complemented by periodic and recurrent assessments of cardiovascular function during and after completion of the treatment course, for early detection and prevention of potentially severe cardiovascular toxicities in patients with MM.

## Introduction

Multiple myeloma (MM) is a neoplastic proliferation of plasma cells in the bone marrow resulting in excessive production of monoclonal immunoglobulin proteins, lytic bone lesions, renal failure, and anemia. It is diagnosed by the presence of one or more myeloma-defining events along with either 10% or more clonal plasma cells in bone marrow or biopsy-proven plasmacytoma [1]. Pharmacotherapy for the management of patients with MM includes drug classes like proteasome inhibitors (PIs), monoclonal antibodies, immunomodulators, alkylating agents, steroids, etc. [2].

Bortezomib (PI) as a chemotherapeutic agent in patients with MM works on the ubiquitin-proteasome system (UPS). UPS is a component of cellular protein homeostasis where damaged or misfolded proteins are tagged by ubiquitin and subsequently broken down by the proteasome. UPS thereby regulates intracellular protein processing and degradation, cell cycle progression, apoptosis, etc. [3]. MM is characterized by dysregulated clonal production of immunoglobulin proteins, many of which are damaged or misfolded. Myeloma cells therefore rely heavily on the UPS system for processing those defective proteins. Inhibition of the proteasome pathway by PIs activates a downstream cascade where damaged proteins accumulate intracellularly in the endoplasmic reticulum, creating endoplasmic reticulum stress and cellular apoptosis. Furthermore, proteasome inhibition also leads to the accumulation of polyubiquitinated substrates, where the ubiquitin required for the process is derived from other cellular substrates like histones. This alters cellular epigenetics, blocks protein translation, and activates alternative degradation pathways and subsequent apoptosis. Lastly, leukemia cells are also known to express higher levels of proteasomes and are thus more susceptible to PIs [3,4]. Cyclophosphamide, an alkylating agent, exerts its cytotoxic effects by cross-linking DNA strands and preventing protein synthesis [5]. Lastly, although dexamethasone is widely used among patients with MM, especially for initial induction therapy prior to hematopoietic stem cell transplant, the mechanism of action behind anti-myeloma therapy is not clearly understood. It is attributed to decreased monoclonal protein synthesis and increased efficacy of other chemotherapeutic regimens used for MM [6].

Cardiovascular toxicity associated with MM therapy has been variably reported among clinical trials and includes a spectrum of adverse events like hypotension, cardiac arrhythmias, and heart failure [7]. The incidence of left ventricular dysfunction associated with bortezomib is reported to be 2-5%, whereas that associated with cyclophosphamide is 7-28% [8]. Here, we present a case of new-onset heart failure with reduced ejection fraction (HFrEF) in a patient with previously normal ejection fraction after treatment with a cyclophosphamide, bortezomib, and dexamethasone (CyBorD) regimen for MM.

## Case presentation

This is the case of a 49-year-old male patient with a past medical history of chronic obstructive pulmonary disease (COPD) from a 17-year history of cigarette smoking, hypertension (HTN) well controlled on a sodium-restricted diet, stage 2 chronic kidney disease, MM on chemotherapy, and major depressive disorder who was brought to the ER due to shortness of breath and altered mental status. The patient was desaturating on a non-rebreather mask with altered mental status at presentation, which led to urgent endotracheal intubation in the ER.

**Figure 1:**
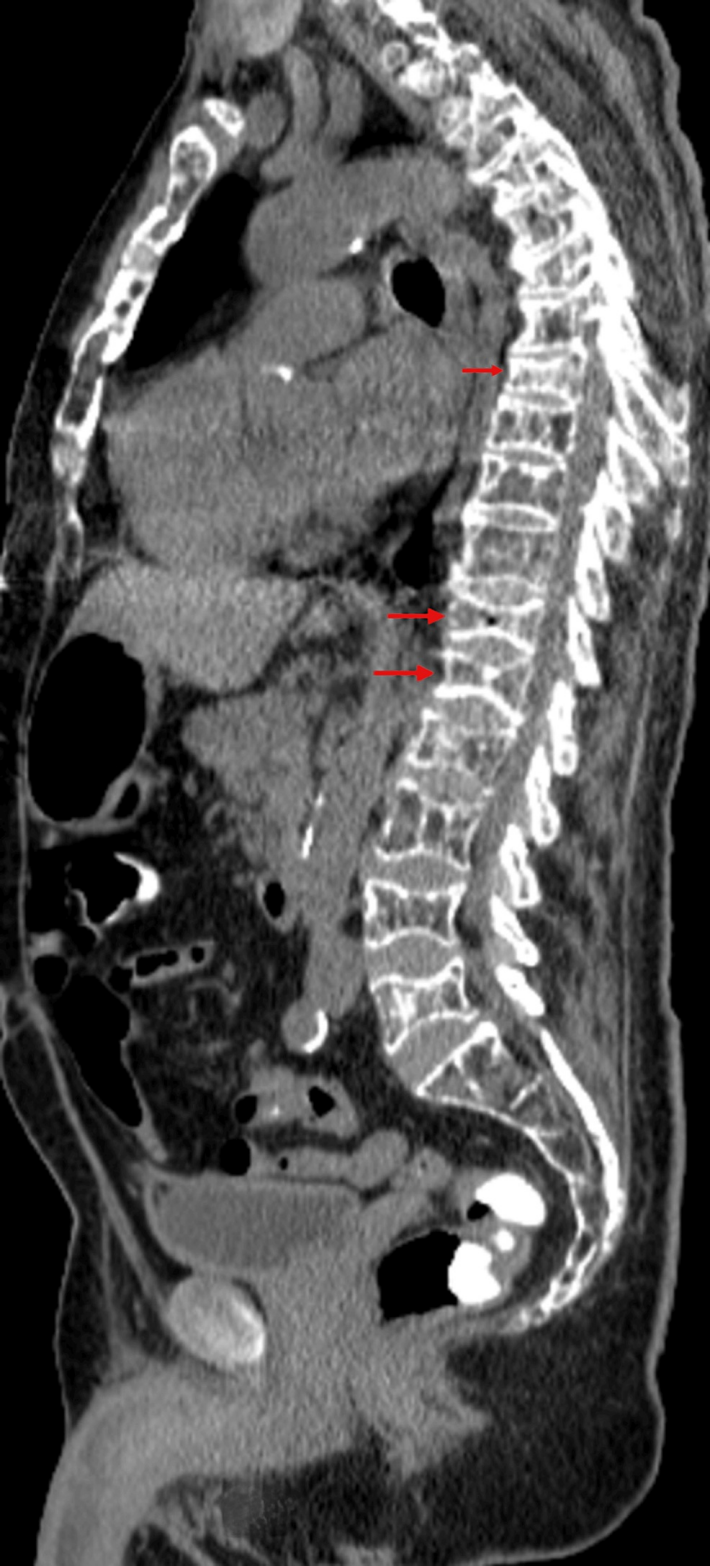
CT scan showing diffuse, severe demineralization of the bones, multiple vertebral body compression fractures (shown by the red arrows), with progressive compression of the T4 vertebral body

The patient had a significant past medical history of MM light chain disease, which was diagnosed during a workup for a 1.7 mm spiculated left lower lobe lung mass in his previous hospitalization for COPD exacerbation. Subsequent follow-up CT scans of the chest three months after initial imaging showed persistence of the mass with a stable size, for which chronic lung scarring was the main consideration. However, new findings showed diffuse, severe demineralization of the bones and multiple vertebral body compression fractures, including T3, T6, T8, T12, and L1, with progressive compression of the T4 vertebral body, typical of metabolic or infiltrative processes (Figure 1). A PET scan was done, which revealed diffuse heterogeneous hypermetabolic activity throughout the bone marrow with intense focal hypermetabolic activity in the right iliac bone. Osteopenia was notable, with multiple lytic lesions in the skeleton and multiple compression fractures. None of the findings were significantly more hypermetabolic than background bone marrow activity (bone marrow standard uptake value 4.3 to 5.8 vs. that of left iliac bone 2.2 to 3.1), which was suggestive of MM. The 1.3 cm nodular opacity in the left lower lobe of the lung was not significantly hypermetabolic.

**Figure 2:**
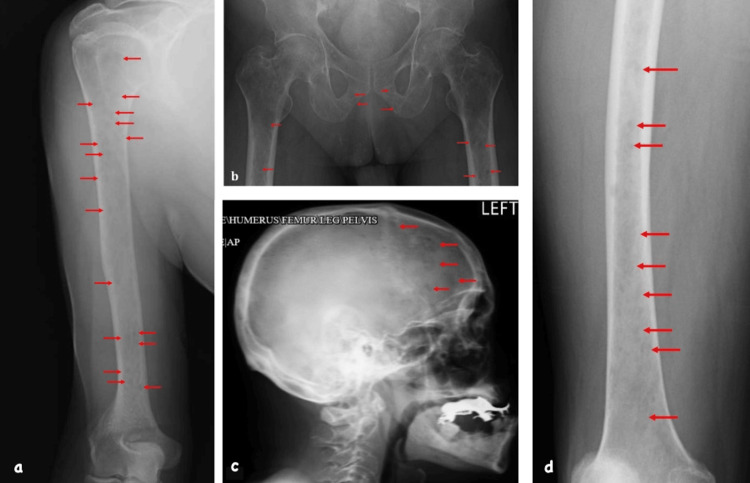
Skeletal survey showing multiple lytic lesions with moth-eaten appearance (red arrows) concerning for MM, seen in the radiographs of the radius (a), pelvis (b), skull (c), and femur (b, d) MM: multiple myeloma

The patient was then lost to follow-up for three months, after which he presented for follow-up again, and a workup for MM was done. Serum protein electrophoresis showed an M spike (0.2%) with the presence of monoclonal free kappa light chains on immunofixation with decreased levels of all immunoglobulins (IgG: 400, IgA: 10, IgM: 10). Serum free light chain quantification showed a kappa to lambda ratio of 1305.81 (free Kappa: 4048 mg/L, free lambda: 3.1 mg/L). A bone marrow biopsy showed a hypercellular marrow (95%), maturing hematopoietic elements with myeloid hyperplasia, and increased atypical plasma cells (20%), consistent with plasma cell neoplasm. No plasmablastic features were noted. Flow cytometry revealed a monoclonal kappa plasma cell (CD38 bright) population (19%). No definite heavy-chain population was noted. Immunohistochemical stains for CD 138 and MUM1 revealed 40-50% plasma cells. Fluorescent in situ hybridization (FISH) oncology for the MM panel was negative for myeloma-related translocations like t(4;14)/FGFR3-IGH rearrangement, t(11;14)/CCND1-IGH rearrangement, and t(14;16)/IGH-MAF rearrangement. Myeloma-related deletions or duplications like 1q, 8q, and 17p1 were also absent. However, small gains were observed on chromosomes 1q, 2p, 6p, 9q, and 16q. In addition, hypodiploidy was present, which may be associated with a more unfavorable prognostic category. A repeat pan-CT showed multiple compression fractures in the thoracolumbar spine. A skeletal survey showed multiple lytic foci on the skull, femurs, pelvis, and humeri corresponding to MM (Figure 2). The patient was diagnosed with a case of MM light chain disease with extensive bone involvement and was identified as high-risk due to the presence of high-risk FISH markers like gain 1q [1]. The patient was not considered transplant-eligible due to frailty (Eastern Cooperative Oncology Group, ECOG performance status of 3).

The patient was planned to start on a quadruplet regimen containing daratumumab (a monoclonal antibody targeting CD38), bortezomib, lenalidomide, and low-dose dexamethasone (DVRd). However, the regimen was held in view of impaired renal function (BUN/Cr: (64 mg/dL)/(1.5 mg/dL) with eGFR of 52 ml/min/1.73 m2). The patient was started on a CyBorD regimen and planned to switch to the DVRd regimen once renal function improved. The patient had a baseline 2D echocardiogram done prior to chemotherapy initiation, which revealed normal findings and an ejection fraction of 55-60%. Left ventricle size, systolic and diastolic functions, and wall thickness were all normal. All the other chambers and valves were also normal. Table 1 below summarizes the chemotherapy sessions that the patient has undergone since the diagnosis of MM.

**Table 1:** Summary of chemotherapy sessions administered CyBorD: cyclophosphamide, bortezomib, and dexamethasone; C: cycle; W: week

Dates	Chemotherapy regimen	Chemotherapy cycles
9/27/2023	CyBorD	C1W1
10/3/2023	CyBorD	C1W2
10/18/2023	Bortezomib	C2W1
10/24/2023	Bortezomib	C2W2
11/7/2023	CyBorD	C3W1
11/21/2023	CyBorD	C4W1
11/28/2023	CyBorD	C4W2
12/12/2023	CyBorD	C5W1
1/3/2024	CyBorD	C6W1
1/10/2024	Cyclophosphamide and bortezomib	C6W2
1/24/2024	Cyclophosphamide and bortezomib	C7W1
1/31/2024	Bortezomib	C7W2
2/14/2024	CyBorD	C8W1

**Figure 3:**
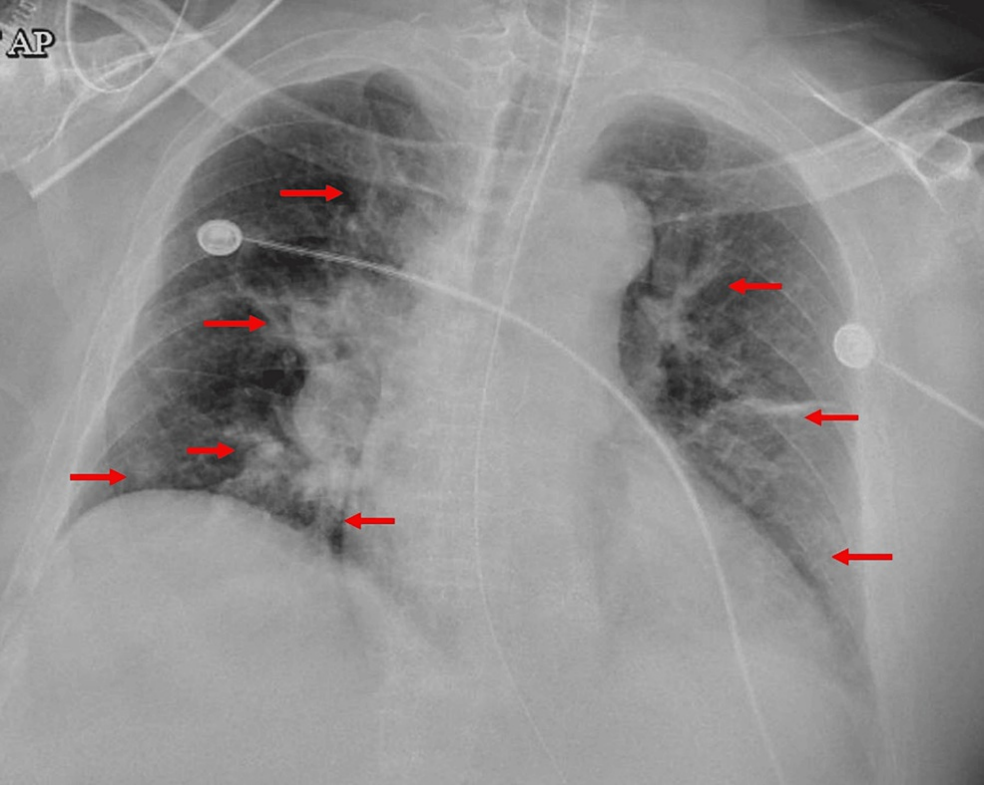
Chest X-ray on admission showing bilateral patchy pulmonary infiltrates (as indicated by red arrows)

**Figure 4:**
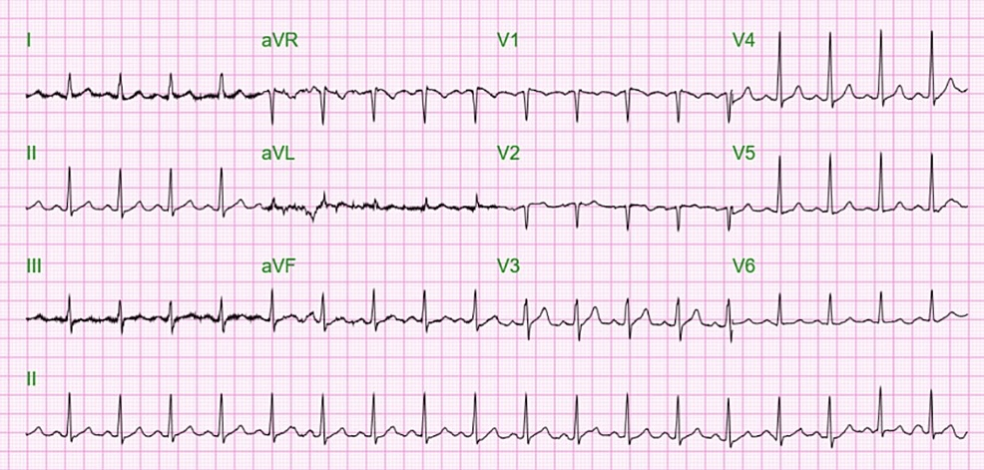
EKG on admission showing sinus tachycardia EKG: electrocardiogram

**Figure 5:**
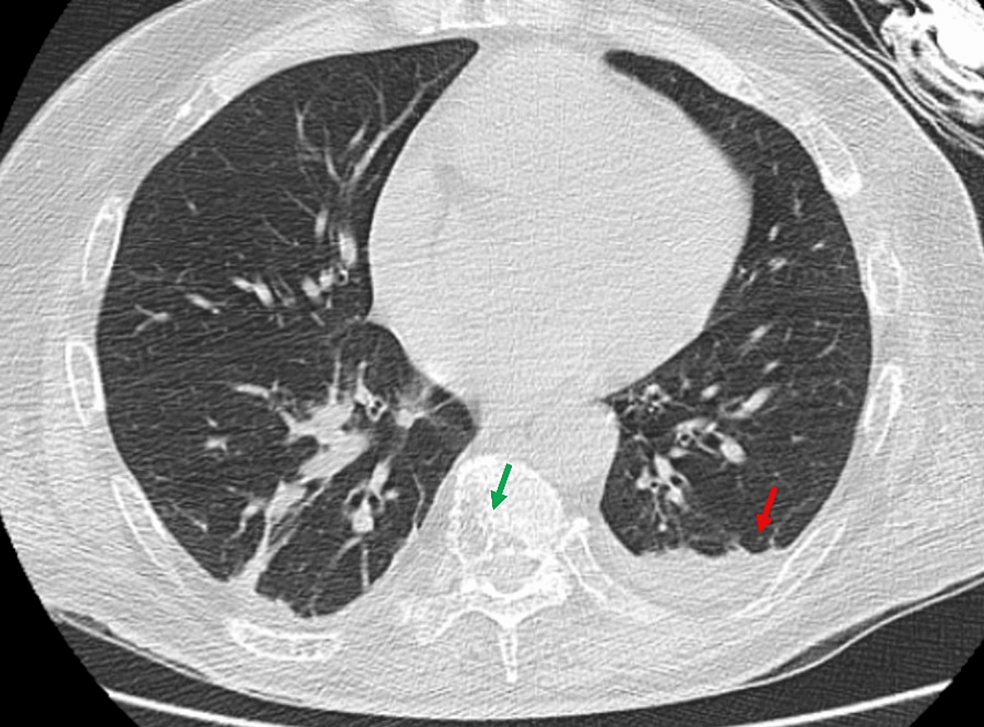
CT scan of the chest showing mild pulmonary edema and minimal pulmonary effusion on the left (red arrow), with bony abnormalities on the thoracic vertebra concerning for MM or metastatic process (green arrow)

**Figure 6:**
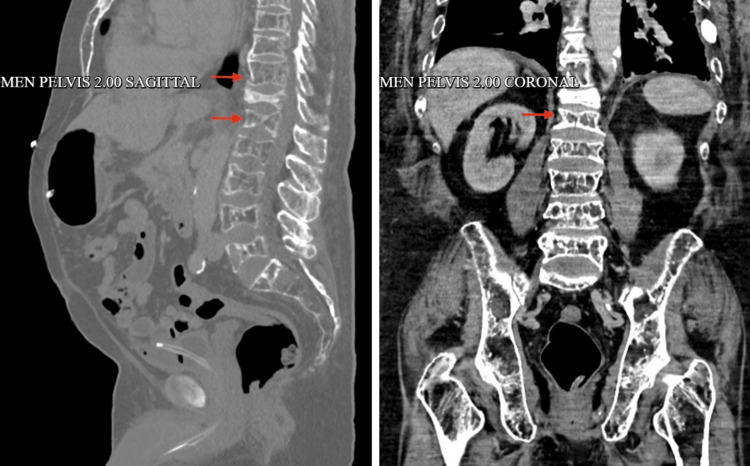
CT scan showing multiple lytic lesions or the thoracolumbar vertebrae with compression fractures and severe demineralization, creating a moth-eaten appearance (red arrows)

After the completion of the eighth cycle of chemotherapy, the patient was brought to the ER severely short of breath with altered mental status, confusion, and palpitations. The patient had a respiratory rate of 30-40 breaths per minute, a heart rate of 120 beats per minute, and a blood pressure of 170/90 mmHg. The chest X-ray showed patchy bilateral pulmonary opacities, likely due to congestion vs. superimposed pneumonia (Figure 3). An electrocardiogram (EKG) showed sinus tachycardia (Figure 4). A CT chest with contrast ruled out pulmonary embolism and reported mild pulmonary edema and minimal pleural effusion on the left (Figure 5). Severe bony demineralization was seen on abdominal and pelvic CT, which supported MM. Multiple stable mild-to-severe lumbar spine compression fractures were also noted (Figure 6).

The echocardiogram done on day one of admission showed normal-sized left and right atria and ventricles. However, the left ventricular systolic function was severely decreased, with an ejection fraction of only 15-20% and grade I diastolic dysfunction. There was global hypokinesis of the left ventricle, although left and right ventricular wall thickness were normal. Right ventricular function was also reduced. To note, the echocardiogram done 4.5 months ago before the initiation of chemotherapy was reported to be normal, as mentioned above. The patient had an elevated brain natriuretic peptide (BNP) of 856 on admission with some troponin leak likely due to demand ischemia (serial high-sensitivity troponin: 87.7, 67.7, and 39.4 pg/ml, normal range: <5-19.7 pg/ml). The patient was treated as a case of exacerbation of new-onset HFrEF likely precipitated by pneumonia, and he responded well to diuresis and antibiotics (five days of meropenem and azithromycin). He was extubated with a good clinical response post-extubation.

Cardiac catheterization is done post-extubation, further confirming a severely depressed left ventricular ejection fraction (20%). However, no angiographic evidence of vessel occlusion or epicardial disease was noted. The patient was started on guideline-directed medical therapy and discharged with a cardiac life vest to follow up in the cardiology and hematology/oncology clinics within one week. Serum immunofixation and serum light chain quantification before initiation of chemotherapy and after completion of four and eight cycles of chemotherapy are tabulated below (Table 2).

**Table 2:** Serum immunofixation and light chain quantification at various time periods IgG: immunoglobulin G; IgA: immunoglobulin A; IgM: immunoglobulin M

Time period	Immunoglobulins quantification on serum immunofixation			Kappa/lambda ratio
	IgG (mg/dL)	IgA (mg/dL)	IgM (mg/dL)	
Before initiation of chemotherapy	400	10	10	1305.81
Status post four cycles of chemotherapy	412	15	16	276
Status post eight cycles of chemotherapy	431	14	15	285

In view of the elevated Kappa/lambda ratio, the patient completed one more chemotherapy session with cyclophosphamide and bortezomib after weighing the risks and benefits of post-discharge oncology clinic follow-up. The patient is planned to get started on the lenalidomide and bortezomib regimen at the next clinic visit, with close monitoring of cardiovascular function with a repeat echocardiogram after one to three months, along with monitoring EKG, BNP, and scheduled cardiology clinic follow-ups.

## Discussion

Bortezomib, a modified dipeptidyl boronic acid analog, has an FDA-approved labeled indication for the treatment of patients with MM and those with mantle cell lymphoma who have received at least one prior therapy [9]. The most frequently reported adverse events with the use of bortezomib include fatigue, malaise, weakness, gastrointestinal adverse events, peripheral neuropathy, thrombocytopenia, neutropenia, and reactivation of the herpes zoster virus [8]. A Phase 3 randomized clinical trial (APEX trial) comparing bortezomib with high-dose dexamethasone among patients with relapsed MM reported a 15% and 13% incidence of cardiac disorders among the bortezomib and dexamethasone groups, respectively. No particular cardiac disorder occurred more than 10% in either group, with incidences of congestive heart failure being similar in both groups (i.e., 2%) [10]. However, heart failure as an adverse event was not reported in two phase 2 open-label clinical trials for bortezomib among patients with relapsed or refractory myeloma [11,12]. In a network meta-analysis of phase 3 randomized clinical trials that explored the cardiotoxic effect of proteasome inhibitors compared to controls, any grade of cardiotoxicity was noted more frequently with PI than the group with no PI (OR: 1.47, 95% CI: 1.19-1.82) among patients with MM. A similar association was seen with high-grade cardiotoxic complications (OR: 1.67, 95% CI: 1.17-2.40). However, the cardiotoxic effect was not shown to be class-specific when PIs were compared against each other, with carfilzomib being the most cardiotoxic among PIs [13]. Isolated case reports are increasingly being reported, citing bortezomib-related congestive heart failure. The presentation ranges from asymptomatic detection during routine ventricular function monitoring to acute symptomatic heart failure episodes with exertional dyspnea, orthopnea, paroxysmal nocturnal dyspnea, bilateral leg swelling, and bibasilar rales [14-16].

Several mechanisms have been proposed to explain the association of bortezomib with cardiovascular toxicity. MM, with a median age of diagnosis of 65 years, is a disease of the elderly population [1]. This translates to the prevalence of higher cardiovascular risk factors in the elderly population, which predisposes the patients to cardiac adverse events secondary to bortezomib. In addition, with advancing age, ubiquitin-proteasome activity is reduced, leading to accentuated proteome instability. Downregulation of proteasomes and transcription factors like NRF1/NRF2, essential for transcriptional regulation of antioxidant and proteostatic genes, is also seen with advancing age [17]. Proteasome function is especially important to maintain the normal size of the heart, preserving the cellular mass and quality of sarcomeres among terminally differentiated myocytes. High metabolic demand in the heart, with increased oxidative phosphorylation and production of reactive oxygen species, creates an unfavorable environment for normal proteostasis [14,17]. The culmination of the above-mentioned factors in the presence of PIs leads to an exaggerated cardiotoxic response and the subsequent development of acute congestive heart failure.

Cardiotoxicity with cyclophosphamide is associated with direct endothelial injury, extravasation of toxic metabolites with interstitial hemorrhage, and direct cytotoxicity to cardiac myocytes. Cyclophosphamide-induced heart failure is reported in 7-28% of cases, especially with high-dose cyclophosphamide used before autologous hematopoietic stem cell transplant [5]. Steroids like dexamethasone are known to cause fluid retention and HTN, leading to an increased cardiac workload [6]. Furthermore, the cumulative cardiotoxic effect of individual components of combination chemotherapy accentuates the cardiovascular toxicity, leading to the development of chemotherapy-induced heart failure [5].

The most common etiologies for HFrEF, or systolic heart failure, include HFrEF secondary to ischemic cardiomyopathy, idiopathic dilated cardiomyopathy, HTN, and valvular disease. Ischemic cardiomyopathy was ruled out in this particular case by the absence of angiographic evidence of epicardial disease in cardiac catheterization, i.e., non-ischemic cardiomyopathy. No evidence of aortic or mitral valve disease was noted in the echocardiogram. The patient’s blood pressure was well controlled without any medication, with a baseline EKG showing no evidence of left ventricular hypertrophy (with normal wall thickness in the echocardiogram), bundle branch block pattern, poor R wave progression, heart block, irregular rhythm, or low limb voltage. Idiopathic dilated cardiomyopathy, cardiac sarcoidosis, or tachycardia-mediated cardiomyopathy were therefore unlikely differentials. The temporal association between initiation of chemotherapy (with a normal echocardiogram) and post-chemotherapy repeat echo shows a reduced ejection fraction, therefore pointing toward CyBorD as a causal factor behind the development of HFrEF.

The treatment approach in patients with MM depends on the patient's eligibility for autologous stem cell transplant and the risk stratification. Triplet regimens like bortezomib, lenalidomide, and dexamethasone (VRd) or daratumumab, lenalidomide, and dexamethasone (DRd) are the current standard regimens for newly diagnosed MM patients unless contraindicated. The daratumumab, lenalidomide, and dexamethasone (DRd) regimen is a reasonable alternative for patients who are not able to tolerate bortezomib [1]. In view of the elevated Kappa/lambda ratio in this case, the patient is planning to get started on the lenalidomide and bortezomib regimen at the next clinic visit, with close monitoring of cardiovascular function with a repeat echocardiogram after one to three months, along with monitoring the EKG, BNP, and scheduled cardiology clinic follow-ups.

With the emerging incidence and reporting of cardiovascular adverse events, it is imperative to perform a cardiovascular risk assessment before initiation of chemotherapy, which includes baseline cardiac evaluation and identification of patients with higher cardiovascular risk. Baseline cardiac evaluation can include assessments of comorbidities and cardiovascular risk factors like smoking, HTN, diabetes, heart failure, coronary artery disease, and renal failure, along with a baseline EKG and echocardiogram. This would help risk stratify patients into those with low risk and high risk for cardiovascular toxicity, which would help devise surveillance strategies during treatment courses along with the need for consultation with cardiology and cardio-oncology specialists. El-Cheikh et al., in a review of cardiac toxicities in MM, have proposed monitoring of ECG, troponin, and BNP every three to six cycles and to consider trans thoracic echocardiograms at least once a year in patients who are treated with bortezomib-based chemotherapy regimens [7].

## Conclusions

Patients undergoing MM chemotherapy can develop cardiovascular adverse events secondary to the effect of chemotherapy and underlying cardiovascular risk factors. It is therefore important to perform a thorough cardiovascular assessment before the initiation of chemotherapy. This should be complemented with periodic and recurrent assessments of cardiovascular function during and after completion of the treatment course for early detection and prevention of potentially severe cardiovascular toxicities.
